# The impact of media use on disparities in physical and mental health among the older people: An empirical analysis from China

**DOI:** 10.3389/fpubh.2022.949062

**Published:** 2022-09-26

**Authors:** Han Wang, Xiaojun Sun, Ruyue Wang, Yang Yang, Yuwei Wang

**Affiliations:** ^1^School of Journalism and Communication, Jinan University, Guangzhou, China; ^2^School of Journalism and Communication, Beijing Sport University, Beijing, China

**Keywords:** health disparities, elderly, media use, education level, health level

## Abstract

**Background:**

The media is playing an increasingly important role in the lives of older adults. Exploring health inequalities in older adults is essential for achieving healthy aging. However, few studies have focused on the effects of different media types on older adults' physical and mental health levels and health inequalities among older adults with varying levels of education from a health communication perspective.

**Objectives:**

The purpose of this study was to investigate the media use, physical and mental health (Self-rated health and subjective well-being) levels of older adults in China, the relationship between different media types use (Traditional media, internet media), and physical and mental health levels and the effects of different media types use on physical and mental health disparities among older adults with varying levels of education.

**Methods:**

The data used in this study are from the 2017 China General Social Survey. The descriptive statistical analysis was conducted on the media use and the health levels of Chinese older adults; analysis of variance and *post hoc* analysis were used to analyze the differences in health levels and frequency of media use among older people with different levels of education; bivariate correlation and regression analyses were conducted to explore the relationship between media use and health levels in older adults; multilevel regression analyses and simple slope plots explored whether the use of different media types widened or narrowed the gap in health levels among older people with varying levels of education.

**Results:**

The results of the study show that (1) the self-rated health levels (M = 2.986, SD = 1.070) are lower in the old people group relative to subjective well-being (M = 3.908, SD = 0.854). While some older adults have mastered the internet media, most of the older population is more accustomed to using traditional media (Especially TV, 77.08% of the elderly are used to watching TV regularly). There are disparities in media use habits and health levels among older adults with different education levels (*p* < 0.01). (2) traditional media use was a significant positive predictor of physical (B = 0.1, *p* < 0.01) and mental health (B = 0.165, *p* < 0.01) in the older age group. Internet media use was a significant positive predictor of physical health (B = 0.052, *p* < 0.01) in the older age group. (3) traditional and internet media use could narrow the physical and mental health disparities between older people with different education levels (*p* < 0.05).

**Conclusions:**

There is an essential correlation between media use and the health levels of old people, and media use can effectively narrow the disparities between the physical and mental health of old people with different educational levels. Society should value the media's important role in promoting older persons' health and well-being. Government-related departments can combine the media with public health campaigns to narrow the health disparity among old people with different educational levels and promote equal healthy aging.

## Introduction

### Population aging and health issues in older age groups

The aging of the population has long been a worldwide concern, and the problem is becoming more and more severe as time goes on. According to the latest data from the World Health Organization, the number and proportion of people aged 60 and over is increasing. In 2019, the number of people aged 60 and overreached 1 billion. By 2030, this number will increase to 1.4 billion and by 2050 to 2.1 billion (1). The growth of the older population is happening at an unprecedented rate, especially in developing countries. As a developing country, China is also amidst a wave of population aging. According to China's seventh population census, the number of people aged 60 and over in China will be 260 million by 2020, accounting for 18.7% of the total population (2). At the same time, China has the fastest recorded growth rate for its old people population (3).

The accelerating aging of the population makes the health of older people a fundamental challenge for society to face. The health problems of old people are not only related to the decline in physical functions and the susceptibility to more physical illnesses (4) but also to mental health. Research has shown that the growth of the older population means an immediate increase in age-related illnesses, for example, dementia, and poor mental health outcomes, such as depression, anxiety, suicide, and severe constraints on the quality of life of older people (5). In addition, health problems in older age groups not only mean greater physical and mental suffering for older people themselves but also hurt other family members (6), directly affecting the rising cost of health care (7), increasing the burden of care on society and the cost of health care (8). Therefore, it is important to pay attention to the physical and mental health of the old people population against the backdrop of the increasingly brutal reality of the aging situation.

### The relationship between media use and health in older age groups

Media use is defined as the public's daily use and exposure to media, including the frequency, duration, type, and attention of certain media (9). According to the trend of media development, the use of media can be divided into the use of traditional media (such as television, radio, newspapers, magazines, etc.) and internet media use (such as computers, networks, etc.) (10). Since media use always starts at a specific point in time and ends at a specific point in time, there are relatively clear starting and ending points and corresponding intervals (11). Therefore, media use can be measured. Retrospective self-reports have been found to have moderately high correlations with other benchmark measures in previous studies, where retrospective self-reports have been the primary measure of media use (11). It provides a prerequisite for measuring the media use behavior of old people.

Along with the continuous development of information technology in society, the influence of media on people's lives is increasing. While most older people are accustomed to using traditional media (12), the growing popularity of the internet access environment has increased the exposure of older people to the internet. According to the 47th Statistical Report on Internet Development in China released by the China Internet Network Information Center, the proportion of old internet users in China reached about 260 million as of December 2020, accounting for 18.4% of the total population (13). As a result, older people enjoy a wide range of media exposure, and both traditional and internet media have made inroads into their life. The number of older people in society has been steadily increasing (14), and so has the interest of communication scholars in the media use of this group. While much research in health communication suggests that media use can influence people's health (15), opinions on the impact of media use on older age groups' health outcomes are varied.

On the one hand, some scholars argue that media use can improve the physical health of older age groups. The use of traditional media such as television, radio, and newspapers, for example, can prevent negative changes in the health-related behavior of older people (16). Not only can internet media effectively improve the physical health of older adults by providing online medical services (17) and health information interventions (18), but internet use has also been shown to significantly increase subjective well-being and life satisfaction, as well as improve the mental health of older adults (12). A study of older adults in China found that internet use may empower them to maintain close intergenerational relationships contributing to their subjective well-being. On the other hand, many other academics have become concerned about the negative impact of media use on older people's physical and mental health. As older people age, limited mobility and geographical distance from relatives make them more likely to feel isolated (19). Because the internet can meet many needs at home, research reveals that it can lead to a loss in offline social activities, leading to less social support for older persons (20) and poor physical and mental health (21). In addition, there is a problem with media content that stereotypes the age of older people, and a study based on the impact of media on older people's mental health showed that such harmful media content could lead to a significant deterioration in older people's mental health, making them more anxious and less calm (22). In addition, there is a problem with media content that stereotypes the age of older people, and a study based on the impact of media on older people's mental health showed that such harmful media content could lead to a significant deterioration in older people's mental health, making them more anxious and less calm (22). Therefore, it is necessary to explore further the relationship between the use of different media types and the physical and mental health of the elderly population and the reasons for the inconsistency with the conclusions of previous studies. We ask questions in this study:

RQ1: What are the associations between different types of media use and older adults' physical and mental health?

### From the “digital divide” to the “health divide” in older age groups

Health communication research has focused not only on the association between media use and health outcomes of older age groups but also on differences in access to and ability to use new information technologies and their further impact on health. Research has shown that older adults experience difficulties adapting to digital lifestyles (23). The digital divide among older people, also known as the silver digital divide, manifests itself in the accessibility of older people's networks and the differences in usage once they are accessed (24). Scholars have used research to reveal possible reasons for the digital divide among older people. On the one hand, age is an important factor influencing older people's media use. Research confirms significant digital divide differences across age groups (25). Lee et al.'s study points to different barriers to digital access and uses for older people at different ages (26). On the other hand, socioeconomic status is also an important factor in determining media access and use. Older age groups are unequal in terms of socioeconomic status (27), and older people with higher education and income levels are more likely to use the internet (28, 29).

This “digital divide” in media use disparities poses several problems for older people daily and even affects their physical and mental health (30). On the one hand, old people persons without access to online technology are unable to obtain timely and accurate health information through internet media and benefit from the ease of online medical services. On the other hand, the technological threshold of the internet disconnects older people from social life, further strengthening their sense of isolation and weakening their sense of social support, thus creating perceived isolation (31). In summary, the digital divide further contributes to the health divide, with differences in media use leading to inequalities in health outcomes.

While the link between differences in socioeconomic status leading to differences in media use and hence health inequalities is well established, whether media use predicts differences in health outcomes between older socioeconomic groups remains to be investigated, and whether different types of media use widen or narrow the health disparities between older socioeconomic groups? The knowledge gap hypothesis offers a possible methodological contribution to addressing this question. The “knowledge gap' hypothesis, proposed by Tichenor in the 1970s, suggested that disseminating media ' information would increase the knowledge gap between people of different socioeconomic statuses (class). People with higher education levels are more capable of acquiring new information than those with lower education levels. With the increased media information over time, people with higher education levels will get more helpful information, extending the “knowledge gap” between the two classes (32). Previous studies have used education level as a measure of socioeconomic status, with domain-specific knowledge as the dependent variable and the product term of education and different types of media use as the independent variable, to predict whether different types of media use can significantly predict the knowledge gap between different educated groups (33, 34). Health inequalities are defined as health disparities systematically related to social advantages/disadvantages such as educational attainment, wealth, and power (35). Socioeconomic status (SES) is an aggregate measure of economic and social status based on factors such as income, education, and occupation (36). It has a 'cumulative effect' on people's health status, i.e., people who are chronically advantaged (or disadvantaged) have better (or worse) levels of health (37). Therefore, in this study, we focus on the impacts of media use on the 'socioeconomic status-health level' link to explore the impacts of media use on health disparities between older people of different socioeconomic status groups. Thus, we raise research questions:

RQ2: Does the use of different media types significantly predict physical and mental health differences in older age groups with different levels of education?
RQ3: If significantly predictable, does media use widen or narrow physical and mental health disparities in older age groups with different levels of education?

## Methods

### Data sources

The data used in this study are from the 2017 China General Social Survey (CGSS). The CGSS is implemented by the National Survey Research Center at Renmin University of China (NSRC) and is the most nationally representative comprehensive social survey project in China. The CGSS survey uses a stratified and staged probability sampling method to choose the sample, covering residents of 31 provinces, autonomous regions, and municipalities directly under the Central Government (excluding Hong Kong Special Administrative Region, Macau Special Administrative Region, and Taiwan). The 2017 CGSS sample data included a valid sample of 12,582. It is worth noting that CGSS 2017 added a new question on residents' use of the internet compared to previously released CGSS data, which provides the basis for this study to investigate the internet media use of the old people population. Due to the differences in health care and living standards in different parts of the world, the age classification of the old people population varies. This study sets the starting age standard for older people at 60 by the provisions in the Law on the Protection of the Rights and Interests of the Old people promulgated by the Chinese government (38). This study selected data related to older people aged 60 years and above in the CGSS survey. After excluding data with missing values and outliers, a final valid sample of 3,878 was obtained.

### Variables selection

#### Dependent variable

In this study, self-rated physical health and subjective well-being were selected as indicators to measure the health of older people, taking into account the existing literature (39). At the physical level, self-rated physical health refers to an individual's subjective personal perception of their overall health (40). It is one of the common indicators used to measure the health status of a population (41). Research has shown that there is consistency between self-rated health and objective health. Self-rated health measures are good indicators of self-assessed health status (42). Therefore, this study used this variable to observe and assess older people's physical health levels. At the psychological level, subjective well-being is an essential indicator of older people's mental health (43). At the psychological level, subjective well-being is an important indicator for measuring the mental health of old people. Subjective well-being is defined as an individual's self-evaluation of their life. Subjective well-being is related to a person's perception and cognition of their quality of life and is considered a fundamental element of positive mental health (44). Self-rated health was measured by a single item respectively. Respondents were asked to reply to the question “what do you think of your current state of physical health,” and the variable in this study was coded 1–5 as “very unhealthy,” “less healthy,” “generally,” “healthy,” and “very healthy.” The five-point Likert-type scale measured subjective well-being for the question: “In general, do you think your life is happy?” with responses including: “1(very unhappy)”, “2(unhappy)”, “3(general)”, “4 (happy)”, “5 (very happy)”.

#### Independent variable

In this paper, we refer to the existing literature (45) and choose education level as an indicator of socioeconomic status. The CGSS 2017 asked for the highest education of the participants. The educational attainment variable in this study was recorded in the range of “Uneducated” (=0), “Primary school” (=1), “Middle school” (=2), “High school/Technical secondary school” (=3), “Junior college” (=4), “Bachelor's degree and above” (=5).

Media use is defined as “the extent to which an audience is exposed to a particular message or type of media content” (46). This study divided media use into traditional media use (newspapers, magazines, radio, television) and internet media use. In the CGSS, participants were asked to report their media use in the past year on a five-point scale, with responses including: “1 (never)”, “2 (rarely)”, “3 (sometimes)”, “4 (often)”, “5 (always).”

#### Control variables

As previous empirical studies have shown that many demographic variables (43, 47, 48) are associated with health levels, several demographic variables were set as control variables in this study. Individual characteristics control variables were as follows: (1) Gender (male coded as 1, female coded as 0). (2) Age. (3) Household registration (Agri-cultural account coded as 1, Non-agricultural account coded as 0). (4) Household income in 2016 (This variable is taken as the natural logarithm, considering that income on fertility intentions may be non-linear, with the squared term of total annual household income added to the regression).

### Data analysis

Data from a total of 3,878 valid samples were used for analysis. We checked for outliers and multicollinearity before analysis and recoded variables and centered scores to fit the study design. Descriptive statistics, analysis of variance (ANOVA), bivariate correlation analysis, *post hoc* analysis, and multilevel regression analysis were used in this study. Descriptive statistics describe older individuals' education, media use, and physical and mental health. ANOVA and *post hoc* analysis were used to analyze the differences in the physical and mental health levels of older people with different levels of education. Bivariate correlation and regression analyses explored associations between media use and mental and physical health in older adults. Multilevel regression analyses with simple slope plots explored whether the use of different media types widened or narrowed the gap in health levels between older people with different levels of education. All statistical analyses were conducted using SPSS 26.0. *P*-values are two-sided, and values < 0.05 were considered statistically significant.

## Results

### Descriptive analysis

The demographic information of the 3,878 samples is shown in Table 1, which reveals that the self-rated health levels (M = 2.986, SD = 1.070) is lower in the old people group relative to subjective well-being (M = 3.908, SD = 0.854).

**Table 1:** Sociodemographic information of the participants (*N* = 3,878).

**Variable**	***N* (%) or Mean ±SD**
**Gender**	
Male	1,910 (49.25)
Female	1,968 (50.75)
**Age (year)**	69.214 ± 7.310
**Total household income in 2016 (CNY)**	
No more than 10,000 yuan	1,039 (26.8)
10,000–30,000 yuan	802 (20.7)
30,001–80,000 yuan	1,287 (33.2)
80,001–150,000 yuan	589 (15.2)
Over 150,000 yuan	161 (4.1)
**Education level**	
Uneducated	901 (23.23)
Primary school	1,270 (32.75)
Middle school	959 (24.73)
High school/Technical secondary school	472 (12.17)
Junior college	163(4.2)
Bachelor's degree and above	113 (2.91)
**Household register**	
Agriculture account	1,983 (51.13)
Non-agricultural account	1,895 (48.87)
**Self-rated health**	2.986 ± 1.070
**Subjective well-being**	3.908 ± 0.854

The media use of the older age group is shown in Table 2. Older people use both paper and broadcast media infrequently but have relatively high access to television, with the combined percentage of often and always choices (77.08%) being the vast majority. Most older people had never used the internet (76.79%), with only 23.21% having access.

**Table 2:** Descriptive statistics of participants' media use (*N* = 3,878).

**Items**	**Never** ***n* (%)**	**Rarely** ***n* (%)**	**Sometimes** ***n* (%)**	**Often** ***n* (%)**	**Always** ***n* (%)**
1. Newspaper media use	2,362 (60.91)	540 (13.92)	331 (8.54)	324 (8.35)	321 (8.28)
2. Magazine media use	2,753 (70.99)	587 (15.14)	289 (7.45)	195 (5.03)	54 (1.39)
3. Broadcast media use	2,329 (60.06)	508 (13.1)	425 (10.96)	369 (9.52)	247 (6.37)
4. Television media use	169 (4.36)	236 (6.09)	484 (12.48)	1,370 (35.33)	1,619 (41.75)
5. Internet media use	2,974 (76.69)	199 (5.13)	156 (4.02)	286 (7.37)	263 (6.78)

The ANOVA results showed significant differences in traditional media use, internet media use, self-rated physical health, and subjective well-being in terms of education level (*p* < 0.01). Table 3 shows the results of the ANOVA for education level and other variables. The frequency of traditional media use (M = 3.11, SD = 0.84), internet media use (M = 3.25, SD = 1.65), self-rated physical health (M = 3.43, SD = 0.92), and subjective well-being (M = 4.05, SD = 0.73) were the highest for the older adults with the highest degree of junior college compared to the other education groups. The frequency of traditional media use (M = 1.80, SD = 0.50), internet media use (M = 1.06, SD = 0.39), self-rated physical health (M = 2.68, SD = 1.07), and subjective well-being (M = 3.73, SD = 0.95) were the lowest for the uneducated older adults compared to the other education groups.

**Table 3:** Results of variance analysis.

	**Education level (Mean** ±**SD)**						**F**	***p***
	**Uneducated** **(*n* = 901)**	**Primary school** **(*n* = 1,270)**	**Middle school** **(*n* = 959)**	**High school/Technical secondary school** **(*n* = 472)**	**Junior college** **(*n* = 163)**	**Bachelor's degree and above** **(*n* = 113)**		
Traditional media use	1.80 ± 0.50	2.13 ± 0.62	2.62 ± 0.79	2.87 ± 0.84	3.11 ± 0.84	3.07 ± 0.87	287.27	*p* < 0.01**
Internet media use	1.06 ± 0.39	1.20 ± 0.72	1.86 ± 1.39	2.49 ± 1.60	3.25 ± 1.65	2.96 ± 1.55	263.67	*p* < 0.01**
Self-rated physical health	2.68 ± 1.07	2.89 ± 1.07	3.13 ± 1.04	3.29 ± 0.99	3.43 ± 0.92	3.35 ± 1.02	38.127	*p* < 0.01**
Subjective well-being	3.73 ± 0.95	3.90 ± 0.85	3.99 ± 0.80	4.01 ± 0.77	4.07 ± 0.73	4.05 ± 0.75	13.002	*p* < 0.01**

**p < 0.01.

It can be found from Table 3 that different education level samples for traditional media use, internet media use, self-rated physical health, and subjective well-being all showed significance (*p* < 0.01), which means the different education level samples for traditional media use, internet media use, physical health, and mental health are different. The *post hoc* analysis needs to be performed. The results of *post hoc* analysis are shown in Table 4. The education level shows a significant level of 0.01 (F = 287.273, *p* < 0.01) for the traditional media use, and the average score comparison results of the groups with significant differences was “Primary school > Uneducated; Middle school > Uneducated; High school/Technical secondary school > Uneducated; Junior college > Uneducated; Bachelor's degree and above> Uneducated; Middle school > Primary school; High school/Technical secondary school > Primary school; Junior college > Primary school; Junior college > Primary school; Bachelor's degree and above > Primary school; High school/Technical secondary school > Middle school; Junior college > Middle school; Bachelor's degree and above > Middle school; Junior college > High school/Technical secondary school; Bachelor's degree and above > High school/Technical secondary school.” The education level shows a significant level of 0.01 (F = 263.67, *p* < 0.01) for internet media use, and the average score comparison results of the groups with significant differences were “Primary school > Uneducated; Middle school > Uneducated; High school/Technical secondary school > Uneducated; Junior college > Uneducated; Bachelor's degree and above > Uneducated; Middle school > Primary school; High school/Technical secondary school > Primary school; Junior college > Primary school; Junior college > Primary school; Bachelor's degree and above > Primary school; High school/Technical secondary school > Middle school; Junior college > Middle school; Bachelor's degree and above > Middle school; Junior college > High school/Technical secondary school; Bachelor's degree and above > High school/Technical secondary school; Junior college > Bachelor's degree and above.” The education level shows a significant level of 0.01 (F = 38.127, *p* < 0.01) for self-rated physical health, and the average score comparison results of the groups with significant differences were “Primary school > Uneducated; Middle school > Uneducated; High school/Technical secondary school > Uneducated; Junior college > Uneducated; Bachelor's degree and above> Uneducated; Middle school > Primary school; High school/Technical secondary school > Primary school; Junior college > Primary school; Junior college > Primary school; Bachelor's degree and above > Primary school; High school/Technical secondary school > Middle school; Junior college > Middle school; Bachelor's degree and above > Middle school.” The education level shows a significant level of 0.01 (F = 13.002, *p* < 0.001) for subjective well-being, and the average score comparison results of the groups with significant differences were “Primary school > Uneducated; Middle school > Uneducated; High school/Technical secondary school > Uneducated; Junior college > Uneducated; Bachelor's degree and above> Uneducated; Middle school > Primary school; High school/Technical secondary school > Primary school; Junior college > Primary school; Junior college > Primary school.”

**Table 4:** Results of *post hoc* analysis.

	**(I)Item**	**(J)Item**	**(I)Mean**	**(J)Mean**	**Difference value (I-J)**	***p***
Traditional media use	0	1	1.799	2.127	−0.328	*p* < 0.01**
	0	2	1.799	2.623	−0.824	*p* < 0.01**
	0	3	1.799	2.867	−1.068	*p* < 0.01**
	0	4	1.799	3.109	−1.31	*p* < 0.01**
	0	5	1.799	3.071	−1.272	*p* < 0.01**
	1	2	2.127	2.623	−0.496	*p* < 0.01**
	1	3	2.127	2.867	−0.739	*p* < 0.01**
	1	4	2.127	3.109	−0.982	*p* < 0.01**
	1	5	2.127	3.071	−0.944	*p* < 0.01**
	2	3	2.623	2.867	−0.243	*p* < 0.01**
	2	4	2.623	3.109	−0.486	*p* < 0.01**
	2	5	2.623	3.071	−0.447	*p* < 0.01**
	3	4	2.867	3.109	−0.242	*p* < 0.01**
	3	5	2.867	3.071	−0.204	*p* < 0.01**
	4	5	3.109	3.071	0.038	0.652
Online media use	0	1	1.059	1.201	−0.142	0.003**
	0	2	1.059	1.86	−0.801	*p* < 0.01**
	0	3	1.059	2.485	−1.426	*p* < 0.01**
	0	4	1.059	3.245	−2.187	*p* < 0.01**
	0	5	1.059	2.956	−1.897	*p* < 0.01**
	1	2	1.201	1.86	−0.659	*p* < 0.01**
	1	3	1.201	2.485	−1.284	*p* < 0.01**
	1	4	1.201	3.245	−2.045	*p* < 0.01**
	1	5	1.201	2.956	−1.755	*p* < 0.01**
	2	3	1.86	2.485	−0.625	*p* < 0.01**
	2	4	1.86	3.245	−1.385	*p* < 0.01**
	2	5	1.86	2.956	−1.095	*p* < 0.01**
	3	4	2.485	3.245	−0.76	*p* < 0.01**
	3	5	2.485	2.956	−0.471	*p* < 0.01**
	4	5	3.245	2.956	0.29	0.029*
Self-rated physical health	0	1	2.677	2.891	−0.214	*p* < 0.01**
	0	2	2.677	3.135	−0.457	*p* < 0.01**
	0	3	2.677	3.288	−0.611	*p* < 0.01**
	0	4	2.677	3.429	−0.752	*p* < 0.01**
	0	5	2.677	3.345	−0.668	*p* < 0.01**
	1	2	2.891	3.135	−0.243	*p* < 0.01**
	1	3	2.891	3.288	−0.397	*p* < 0.01**
	1	4	2.891	3.429	−0.538	*p* < 0.01**
	1	5	2.891	3.345	−0.454	*p* < 0.01**
	2	3	3.135	3.288	−0.154	0.009**
	2	4	3.135	3.429	−0.295	0.001**
	2	5	3.135	3.345	−0.211	0.043*
	3	4	3.288	3.429	−0.141	0.137
	3	5	3.288	3.345	−0.057	0.603
	4	5	3.429	3.345	0.084	0.51
Subjective well-being	0	1	3.731	3.897	−0.165	*p* < 0.01**
	0	2	3.731	3.991	−0.259	*p* < 0.01**
	0	3	3.731	4.015	−0.283	*p* < 0.01**
	0	4	3.731	4.067	−0.336	*p* < 0.01**
	0	5	3.731	4.053	−0.322	*p* < 0.01**
	1	2	3.897	3.991	−0.094	0.010**
	1	3	3.897	4.015	−0.118	0.010**
	1	4	3.897	4.067	−0.171	0.016*
	1	5	3.897	4.053	−0.156	0.061
	2	3	3.991	4.015	−0.024	0.611
	2	4	3.991	4.067	−0.077	0.285
	2	5	3.991	4.053	−0.062	0.459
	3	4	4.015	4.067	−0.053	0.494
	3	5	4.015	4.053	−0.038	0.666
	4	5	4.067	4.053	0.014	0.89

*p < 0.05; **p < 0.01, 0 = Uneducated, 1 = Primary school, 2 = Middle school, 3 = High school/Technical secondary school, 4 = Junior college, 5 = Bachelor's degree and above.

### Preliminary analysis

Bivariate correlation analysis (Table 5) showed that there was a significant positive correlation (*p* < 0.01) between the level of education, frequency of traditional media use, frequency of internet media use, and self-rated physical health. Bivariate correlation analysis (Table 6) showed that there was a significant positive correlation between education level, frequency of traditional media use, frequency of internet media use, and subjective well-being (*p* < 0.01).

**Table 5:** Bivariate correlation between self-rated health and other variables.

	**Self-rated** **health**	**Age**	**Gender** **(male)**	**Household register** **(Agriculture)**	**Total household** **income**	**Education** **level**	**Traditional** **media use**
Age	−0.052**						
Gender (male)	0.098**	−0.007					
Household register (Agriculture)	−0.174**	−0.085**	0.017				
Total household income	0.248**	−0.036*	−0.014	−0.549**			
Education level	0.210**	−0.097**	0.202**	−0.519**	0.445**		
Traditional media use	0.200**	0.03	0.117**	−0.467**	0.401**	0.504**	
Internet media use	0.183**	−0.188**	0.056**	−0.390**	0.373**	0.485**	0.327**

*p < 0.05; **p < 0.01.

**Table 6:** Bivariate correlation between subjective well-being and other variables.

	**Subjective** **well-being**	**Age**	**Gender** **(male)**	**Household register** **(Agriculture)**	**Total household** **income**	**Education** **level**	**Traditional** **media use**
Age	0.075**						
Gender (male)	−0.017	−0.007					
Household register (Agriculture)	−0.127**	−0.085**	0.017				
Total household income	0.175**	−0.036*	−0.014	−0.549**			
Education level	0.116**	−0.097**	0.202**	−0.519**	0.445**		
Traditional media use	0.201**	0.03	0.117**	−0.467**	0.401**	0.504**	
Internet media use	0.073**	−0.188**	0.056**	−0.390**	0.373**	0.485**	0.327**

*p < 0.05; **p < 0.01.

Further linear regression analysis was conducted based on bivariate correlation analysis (see Table 7). Model 1, with self-rated physical health as the dependent variable, explained 8.5% of the total variance in the outcome variable. Model 2, with subjective well-being as the dependent variable, explained 5.8% of the outcome variable. The linear regression results for Model 1 indicated that education level (B = 0.036, *P* < 0.05), frequency of traditional media use (B = 0.1, *P* < 0.01), and frequency of internet media use (B = 0.052, *P* < 0.01), were significant positive predictors of self-rated physical health in the older age group. The linear regression results of Model 2 indicated that the frequency of traditional media use (B = 0.165, *P* < 0.01) was a significant positive predictor of the subjective well-being of older people.

**Table 7:** Linear regression analysis results.

	**Model 1**		**Model 2**	
	**B**	**SE**	**B**	**SE**
Constant	1.98**	0.207	2.427**	0.168
Age	0.161**	0.035	−0.076**	0.028
Gender (male)	−0.004	0.002	0.009**	0.002
Household register (Agriculture)	0.043	0.045	0.068	0.037
Total household income	0.038**	0.004	0.022**	0.003
Education level	0.036*	0.018	0.01	0.015
Traditional media use	0.1**	0.025	0.165**	0.02
Internet media use	0.052**	0.016	−0.002	0.013
*R* ^2^	0.085		0.058	
Adjusted *R*^2^	0.084		0.056	
F	F (7,3706) = 49.463, p = 0.000		F (7,3706) = 32.615, p = 0.000	
Dependent variable	Self-rated health		Subjective well-being	

*p < 0.05; **p < 0.01.

### Impact of media use on physical and mental health disparities

To examine whether different types of media use widen or narrow the physical and mental health gap among older people with different levels of education. This study examined the effect of different types of media use on the 'education level-physical and mental health link through multilevel regression analysis using an interaction effects model. Thus, Table 8 shows the multilevel regression model “a” when the interaction variable is Education^*^Frequency of traditional media use, and the dependent variable is self-rated physical health. Table 9 shows the multilevel regression model “b” when the interaction variable is Education^*^Frequency of internet media use, and the dependent variable is self-rated physical health. Table 10 shows the multilevel regression model “c” when the interaction variable is Education^*^Frequency of traditional media use, and the dependent variable is subjective well-being. Table 11 shows the multilevel regression model “d” when the interaction variable is Education^*^Frequency of internet media use, and the dependent variable is subjective well-being. The multilevel regression model is performed as follows: first, the independent variables are mean-centered; second, the control variable, educational attainment, is entered into block 1; second, media use is entered into block 2; and finally, the interaction variable is entered into block 3.

**Table 8:** Results of hierarchical regression analysis a.

	**Block 1**		**Block 2**		**Block 3**	
	**B**	**SE**	**B**	**SE**	**B**	**SE**
Constant	2.406**	0.196	2.481**	0.196	2.441**	0.196
Gender (male)	0.169**	0.035	0.159**	0.035	0.145**	0.035
Age	−0.005*	0.002	−0.005*	0.002	−0.004	0.002
Household register (Agriculture)	−0.014	0.044	0.023	0.045	0.048	0.045
Total household income	0.042**	0.004	0.04**	0.004	0.04**	0.004
Education level	0.074**	0.017	0.052**	0.017	0.065**	0.018
Traditional media use			0.105**	0.025	0.132**	0.026
Education*Traditional media use					−0.069**	0.017
*R* ^2^	0.078		0.083		0.087	
adjust *R*^2^	0.077		0.081		0.085	
F	F _(5, 3, 708)_ = 63.047, *p* = 0.000		F_(6, 3, 707)_ = 55.715, *p* = 0.000		F_(7, 3, 706)_ = 50.450, *p* = 0.000	
Dependent variable	Self-rated health					

*p < 0.05; **p < 0.01.

**Table 9:** Results of hierarchical regression analysis b.

	**Block 1**		**Block 2**		**Block 3**	
	**B**	**SE**	**B**	**SE**	**B**	**SE**
Constant	2.403**	0.196	2.312**	0.197	2.306**	0.197
Gender (male)	0.169**	0.035	0.17**	0.035	0.164**	0.035
Age	−0.005*	0.002	−0.003	0.002	−0.003	0.002
Household register (Agriculture)	−0.014	0.044	0.009	0.044	0.025	0.045
Total household income	0.042**	0.004	0.041**	0.004	0.04**	0.004
Education level	0.074**	0.017	0.055**	0.017	0.062**	0.018
Internet media use			0.056**	0.016	0.087**	0.019
Education*Internet media use				−0.031**	0.011
*R* ^2^	0.078		0.081		0.083	
adjust *R*^2^	0.077		0.08		0.082	
F	F _(5, 3, 708)_ = 63.047, *p* = 0.000		F _(6, 3.707)_ = 54.813, *p* = 0.000		F _(7, 3, 706)_ = 48.087, *p* = 0.000	
Dependent variable	Self-rated health					

*p < 0.05; **p < 0.01.

**Table 10:** Results of hierarchical regression analysis c.

	**Block 1**		**Block 2**		**Block 3**	
	**B**	**SE**	**B**	**SE**	**B**	**SE**
Constant	2.715**	0.16	2.83**	0.159	2.787**	0.159
Gender (male)	−0.061*	0.028	−0.076**	0.028	−0.092**	0.028
Age	0.01**	0.002	0.009**	0.002	0.011**	0.002
Household register (Agriculture)	0.011	0.036	0.069	0.036	0.094**	0.036
Total household income	0.025**	0.003	0.022**	0.003	0.021**	0.003
Education level	0.043**	0.014	0.009	0.014	0.023	0.014
Traditional media use			0.165**	0.02	0.196**	0.021
education*Traditional media use				−0.072**	0.013
*R* ^2^	0.041		0.058		0.083	
Adjust *R*^2^	0.04		0.056		0.082	
F	F _(5, 3, 708)_ = 31.899, *p* = 0.000		F _(6, 3, 707)_ = 38.057, *p* = 0.000		F _(7, 3, 706)_ = 48.087, *p* = 0.000	
Dependent variable	Subjective well-being					

*p < 0.05; **p < 0.01.

**Table 11:** Results of hierarchical regression analysis d.

	**Block 1**		**Block 2**		**Block 3**	
	**B**	**SE**	**B**	**SE**	**B**	**SE**
Constant	2.715**	0.16	2.708**	0.161	2.705**	0.161
Gender (male)	−0.061*	0.028	−0.061*	0.028	−0.065*	0.028
Age	0.01**	0.002	0.011**	0.002	0.011**	0.002
Household register (Agriculture)	0.011	0.036	0.013	0.036	0.022	0.037
Total household income	0.025**	0.003	0.025**	0.003	0.025**	0.003
Education level	0.043**	0.014	0.042**	0.014	0.045**	0.014
Internet media use			0.004	0.013	0.023	0.016
education*Internet media use				−0.019*	0.009
*R* ^2^	0.041		0.041		0.042	
Adjust *R*^2^	0.04		0.04		0.04	
F	F _(5, 3, 708)_ = 31.899, *p* = 0.000		F _(6, 3, 707)_ = 26.593, *p* = 0.000		F _(7, 3, 706)_ = 23.374, *p* = 0.000	
Dependent variable	Subjective well-being					

*p < 0.05; **p < 0.01.

The results in Table 8 show that the product term of education level and frequency of traditional media use is significant. The frequency of traditional media uses significantly predicts physical health disparities due to different levels of education.

**Figure 1:**
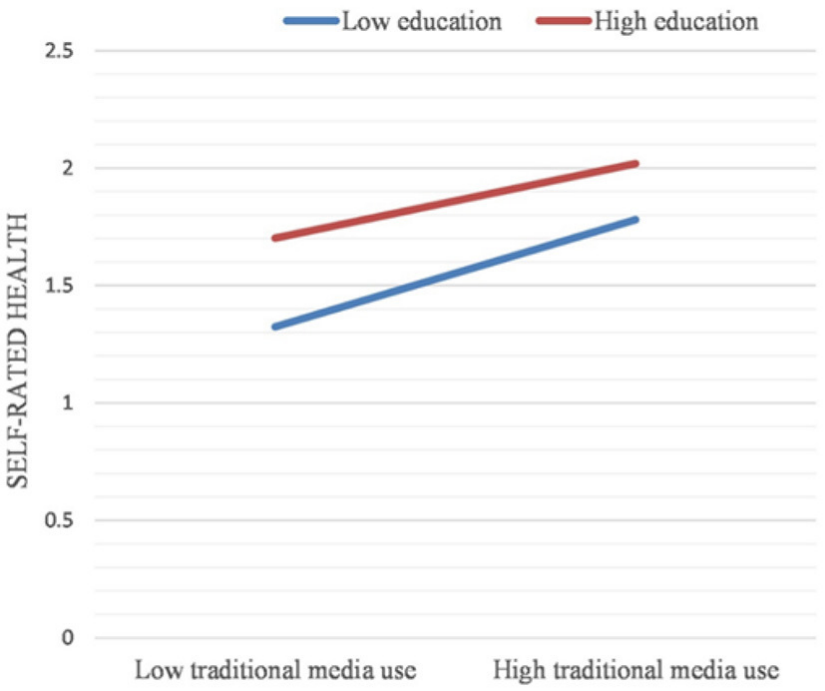
Simple slope plot for the interaction between traditional media use and education (Dependent variables: Self-rated health).

The effect of the frequency of traditional media use on the physical health disparities due to different levels of education is further shown by plotting a simple slope diagram (see Figure 1). The increased frequency of traditional media uses further narrowed the physical health disparities between the highly educated old people group and the less educated old people group. In other words, the greater the use of traditional media, the smaller the physical health disparities between older adults with higher education and those with lower education (B = −0.069, *p* < 0.01).

The results in Table 9 show that the product term of education level and frequency of internet media use is significant. The frequency of internet media use is a significant predictor of physical health disparities due to different levels of education.

**Figure 2:**
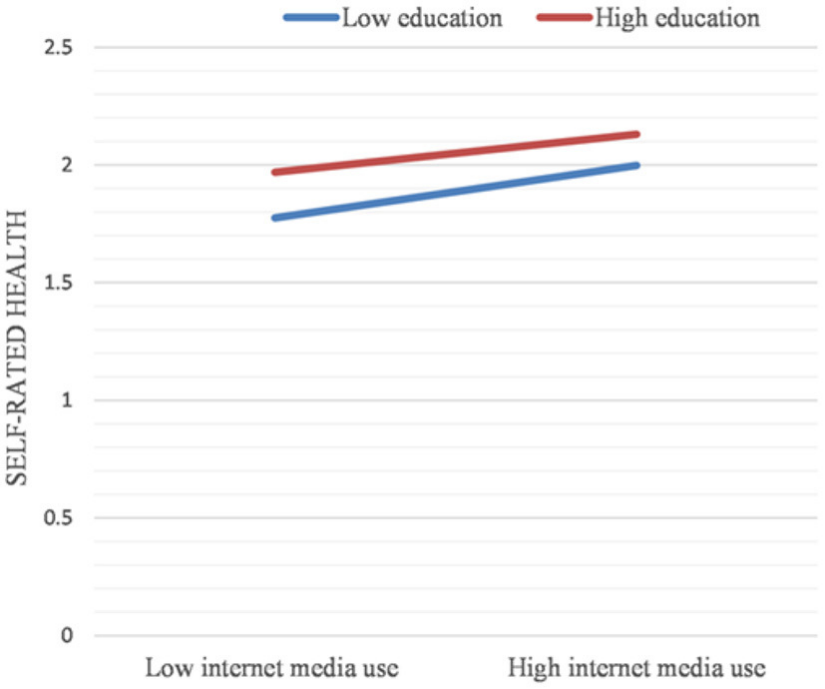
Simple slope plot for the interaction between internet media use and education (Dependent variables: Self-rated health).

The effect of internet media use frequency on the physical health disparities due to different levels of education is further shown by plotting a simple slope graph (see Figure 2). The increased frequency of internet media uses further narrowed the physical health disparities between the highly educated older adult group and the less educated older adult group. In other words, the greater the use of internet media, the smaller the physical health disparities between older adults with higher education and those with lower education (B = −0.031, *p* < 0.01).

The results in Table 10 show that the product term of education level and frequency of traditional media use is significant. The frequency of traditional media uses significantly predicts mental health disparities due to different levels of education.

**Figure 3:**
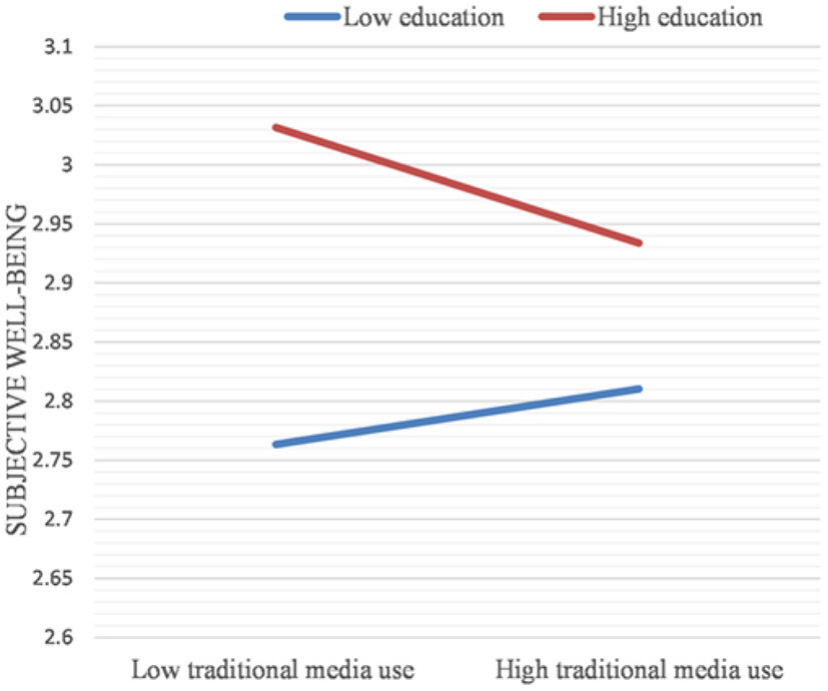
Simple slope plot for the interaction between traditional media use and education (Dependent variables: Subjective well-being).

The effect of the frequency of traditional media use on the mental health disparities due to different levels of education is further shown by plotting a simple slope graph (see Figure 3). The increased frequency of traditional media uses further narrowed the mental health disparities between the highly educated older adult group and the less educated older adult group. In other words, the greater the use of traditional media, the smaller the mental health disparities between older adults with higher education and those with lower education (B = −0.072, *p* < 0.01).

The results in Table 11 show that the product term of education level and frequency of internet media use is significant. The frequency of internet media use significantly predicts mental health disparities due to different levels of education.

**Figure 4:**
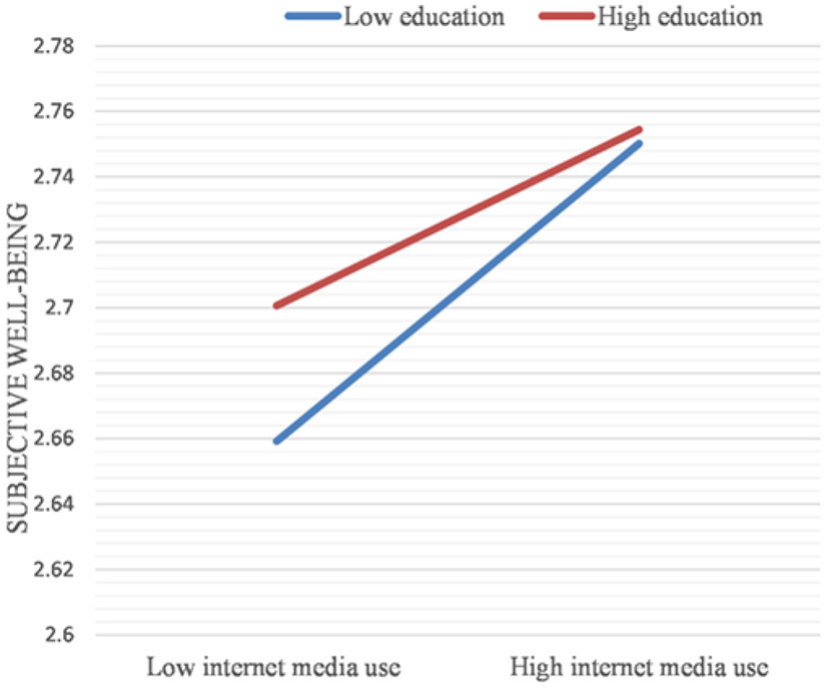
Simple slope plot for the interaction between internet media use and education (Dependent variables: Subjective well-being).

The effect of internet media use frequency on mental health disparities due to different levels of education is further shown by plotting a simple slope graph (see Figure 4). The increased frequency of internet media uses further narrowed the mental health disparities between the highly educated older adult group and the less educated older adult group. In other words, the greater the use of internet media, the smaller the mental health disparities between older adults with higher education and older adults with lower education (B = −0.019, *p* < 0.05).

## Discussion

Based on nationally representative data, this paper investigated the use of different media types among old people, the correlation between the use of different media types and physical and mental health, and the impact of different media type use on physical and mental health disparities among elderly groups with different educational backgrounds. Furthermore, the health disparity problem was displayed through a simple slope graph. We found an important correlation between media use and health in older adults based on the results. Specifically, traditional media use was an important indicator that significantly and positively predicted the physical and mental health of the old people, while internet media could only positively predict the physical health of the old people. In addition, traditional and internet media use could significantly predict physical and mental health disparities among old people with different educational backgrounds. Both traditional media use and internet use media have narrowed the physical and mental health disparities between elderly groups with different educational backgrounds. Finally, we used our findings and available literature to make recommendations for closing health disparities in old people and promoting health and well-being in the elderly population.

### Media use and the health of older people

Firstly, we investigated media use among older adults. The media usage of the old people in Table 2 is consistent with the existing research results. Although some older people have mastered internet media, most elderly groups are more accustomed to using traditional media. However, literature of recent years has focused on the use of internet media and social media and the consequences of media use for old people (49, 50), and most of the research on traditional media use by old people is still carried out in the last century (51). The results of this study show that in the current media environment, traditional media still plays an essential role in the lifestyle of old people. Therefore, whether it is traditional or internet media, we suggest that future research needs to analyze the media use behavior of old people in the current media environment more comprehensively.

Secondly, this study found that traditional media use was a significant positive predictor of physical and mental health in the older age group, consistent with previous research findings (16). Media use behavior primarily reflects the socioeconomic status of users (52). However, due to the relative popularity of traditional media use among old people (Table 2), we speculate that this may be related to the social function of mass communication. The traditional media have proven to be very useful in effectively communicating health information (53), and public health organizations can use traditional media to produce media content around health topics (54). In China, the credibility of traditional media is higher than that of social media (55), making it easier for most older people who use traditional media (12) to trust health information in traditional media. As media content affects people's perceptions, which can influence their attitudes and behaviors (56), thus traditional media use was a significant positive predictor of physical and mental health in the older age group.

In addition, this study also found that internet media use was an important indicator of a significantly positive prediction of the physical health of old people, which is also consistent with previous research conclusions. Previous research has found a significant positive correlation between the socioeconomic status of older adults and the frequency of Internet use (57). Research on the sociodemographic correlations of ICT (information and communications technology) use among older adults showed that the average user is younger, more educated, and has a higher income than the average non-user (58–60). The analysis of variance in this study (Table 3) also reflected this to some extent. The economic productivity and physical strength of older adults decline with age (61), and the association between socioeconomic status (SES) and physical health is fairly robust (62). Therefore, the frequency of internet media usage is one of the indicators reflecting socioeconomic status (52). Compared with the old people with lower socioeconomic status, the old people with higher socioeconomic status have a higher frequency of internet media use, and their self-rated physical health level is also higher. In this study, internet media use did not significantly predict mental health in older age groups. We offer two possible explanations for this. First, conclusions about the benefits of internet media use on older adults' mental health and well-being may be premature or overstated, consistent with established conclusions. For example, the results of a meta-analysis showed that computer or internet training did not affect the well-being of older adults (63). Second, we believe that assertions that socioeconomic status significantly positively affects older adults' mental health may be premature. It is reflected in some studies' findings that there is no significant association between socioeconomic status and depression in older adults (64, 65). Therefore, although their socioeconomic status reflects the internet use behavior of the old people, the internet use frequency of the old people cannot positively and significantly predict their mental health.

### The role of media use on physical and mental health disparities in older age groups

Another finding of this study was that traditional and internet media use significantly predicted the physical and mental health disparities between older people with different education levels. Traditional and internet media use narrowed the physical and mental health disparities between older people with different education levels. The ceiling effect theory offers the possibility of explaining that traditional media use can narrow health disparities in older age groups. The ceiling effect theory states that there is no end to the individual's quest for specific knowledge, and once a particular “ceiling” is reached, the knowledge increase slows down or stops. Those with higher socioeconomic status acquire knowledge faster and reach their “ceiling” sooner; those with lower economic status increase their knowledge more slowly but eventually catch up with the former over time (66). Access to media information affects people's perceptions, which can influence attitudes and behaviors (67). There may also be a “ceiling effect” on access to health knowledge for older people with different education levels. Therefore, traditional media use can ultimately positively impact narrowing the physical and mental health disparities. Internet media plays a massive role in popularizing public health information for health information. The low-barrier nature of internet media allows health professionals and laypeople alike to produce health content on online platforms and use the internet to read, comment, and share it globally with the public through the internet (68). As a result, different educational groups have access to diverse health knowledge and ideas *via* the internet, explaining why internet media can narrow the physical and mental health disparities among older people. Social media has narrowed social inequalities caused by organizational size and social status (69). This study provides further evidence of the positive impact of internet media on narrowing health disparities in older age groups.

### Recommendations for narrowing the physical and mental health disparities in older age groups

This study confirmed that traditional and internet media use significantly predicted physical and mental health disparities in older age groups with different education levels, so it is necessary to make recommendations for promoting equal healthy aging.

Firstly, the mass media plays a huge role in disseminating health information to the public regarding prevention, health risk reduction, and provision of medication (70). A study with traditional media pointed out that repeated coverage of the same topic increases the likelihood that media exposure to inactive people will eventually lead to that information (71). Therefore, traditional media should repeatedly promote important health issues relevant to older people so that health information is more equally accessible to different socioeconomic groups, reducing the health disparities between older people with different education levels.

Secondly, integrating the media with public health campaigns is also very important to promote the health and well-being of older adults. The main purpose of public health campaigns is to influence the behavior of individuals by recommending changes in their habits and encouraging preventive behaviors (72). Moreover, mass media campaigns have long been a tool for promoting public health (73). Government-related departments and public health organizations can leverage social media and its low-cost operation and capacity to increase campaign reach, making it a potential communication channel for equitable, healthy aging campaigns (74). In addition, forms such as community-organizing strategies, Internet-based education, and mass media advertising (75) can all be used for reference in the health promotion of old people.

Thirdly, as discussed earlier, the digital divide further contributes to creating a health divide, so bridging the digital divide in older age groups is important in reducing the health disparities between older age groups with different education levels. “Digital feedback” refers to the teaching of older generations by younger generations in terms of digital access, use, and literacy (76), which has proven to be an important factor in helping older groups to be able to use internet media (77). Therefore, it is necessary to encourage and create a family culture of “digital feedback” so that children are aware of the benefits of digital skills for older people, which will also enable more older people to use internet media to access health information and thus narrow the “digital divide”. In addition, online emotional support is an essential factor contributing to older people's mental health, especially as the COVID-19 global pandemic continues, and strict social distancing measures may negatively impact access to emotional support in older age groups which in turn may affect health levels. Therefore, this study recommends that, on the one hand, individual disparities among older people from different socioeconomic status groups need to be taken into account. Different internet media education interventions need to be developed for older people from different backgrounds (age, gender, and education level) to help them improve their digital competence. On the other hand, not only is there a need to raise awareness of the need for younger generations to educate older generations about reverse digital education, but society and the children in the family also need to help older groups access effective online emotional support through internet media to promote their mental health.

Finally, evaluating the success of a public health campaign is critical. It helps policymakers to improve strategies and close existing gaps (78). Therefore, it is also important to evaluate the effects of health-promoting campaigns on older adults. Referring to the practice of Rafael Pinto et al. (79), we recommend that government-related departments and public health organizations develop the collection and evaluation of relevant media data so that decision-makers can obtain this information clearly and accurately, to promote health literacy education, media literacy education, and healthy aging strategies for the old people.

## Conclusion

Based on a nationally representative sample of the CGSS 2017, this study examined the association between the frequency of different media use and older adults' physical and mental health. This paper used an interaction effects model combined with simple slope plots to explore whether different media types of use significantly predicted physical and mental health disparities in older adults at different levels of education. The findings suggested an essential correlation between media use and fitness levels. Traditional media use was a significant positive predictor of physical and mental health in the older age group. In comparison, internet media use only was a significant positive predictor of physical health in the older age group. Traditional and internet media use narrowed the physical and mental health disparities between older age groups with different education levels. This study draws on knowledge gap-related research to demonstrate the role of media use in reducing physical and mental health disparities among older people with different education levels. The findings of this study could provide recommendations for enhancing the health and well-being of older age groups and provide a reference point for promoting equal healthy aging from a media perspective.

## Implications and limitations

This study has specific theoretical and practical implications. Firstly, with the help of the research on the knowledge gap, this study examined the physical and mental health disparities in older age groups and demonstrated the role of media use in reducing the physical and mental health disparities in older age groups with different education levels, which provides a reference point for promoting equal healthy aging. Secondly, with the acceleration of the aging process, the health of older people is a significant public health issue worldwide. An empirical study on a nationally representative sample of older people in China can provide an idea for promoting older people's health and addressing older people's health inequalities, which has some social significance.

In addition, there are some limitations to this study. Firstly, the measurement of socioeconomic status is very complex, and our use of the education levels variable to measure socioeconomic status, while feasible, still has limitations. Future data analysis could use a more comprehensive measure of socioeconomic status. Secondly, this study used secondary data with limited items, so we only included older people's education levels and media use as important influences on physical and mental health. Future studies could include more other influences. In addition, chronic health conditions and disability were not accounted for in the CGSS, so we could not include them as control variables in the analysis. Future research could avoid this limitation by collecting primary data. Finally, the cross-sectional data only allow possible interpretations of the causal relationship between media use and physical and mental health disparities. Therefore, future panel studies or supplementary data are recommended to assess the causal impact of media use on physical and mental health disparities.

## Data availability statement

Publicly available datasets were analyzed in this study. This data can be found at: http://cgss.ruc.edu.cn/English/Home.htm.

## Ethics statement

The studies involving human participants were reviewed and approved by the Institutional Review Board of Social Sciences and Humanities of Jinan University. Written informed consent for participation was not required for this study in accordance with the national legislation and the institutional requirements.

## Author contributions

HW and XS designed the study and analyzed the data. HW, RW, and YY were involved in manuscript writing. YW revised the manuscript. All authors have read and approved the manuscript.

## Conflict of interest

The authors declare that the research was conducted in the absence of any commercial or financial relationships that could be construed as a potential conflict of interest.

## Publisher's note

All claims expressed in this article are solely those of the authors and do not necessarily represent those of their affiliated organizations, or those of the publisher, the editors and the reviewers. Any product that may be evaluated in this article, or claim that may be made by its manufacturer, is not guaranteed or endorsed by the publisher.
